# Traditional detoxification of wild yam (*Dioscorea hispida* Dennst) tuber in chips processing at East Java, Indonesia

**DOI:** 10.1186/s42779-022-00164-1

**Published:** 2022-12-19

**Authors:** Teti Estiasih, Kgs. Ahmadi, Irawati Nur Indah Sari, Dessy Eka Kuliahsari, Erryana Martati

**Affiliations:** 1 Department of Food Science and Technology, Faculty of Agricultural Technology, Universitas Brawijaya, Malang, Indonesia; 2 Department of Agro-Industrial Technology, Faculty of Agricultural, Tribhuwana Tunggadewi University, Malang, Indonesia; 3 Master Program of Agricultural Product Technology, Faculty of Agricultural Technology, Universitas Brawijaya, Malang, Indonesia

**Keywords:** Acetone cyanohydrin, Cyanogenic glycoside, Detoxification, *Dioscorea*, HCN, Wild yam

## Abstract

*Dioscoreaceae* or yam is a family of tuber that comprises many members with variability in utilization and their intensity of consumption. This family has wide variability and is used not only as food but also for medical purposes due to their bioactive compounds. One of *the Dioscoreaceae* family is wild yam (*Dioscorea hispida* Dennst), rich in carbohydrates but has an obstacle of high cyanide level. Historically, along with cassava, wild yam is the staple food in some places in Indonesia. There is a long history of traditional detoxification methods of wild yam with slightly different steps among different places. The shifting of staple food to rice excludes wild yam consumption. One of the remaining products from wild yam is chips. Wild yam chips are a traditional snack that is also produced by traditional detoxification. This paper is aimed to review the scientific basis for each step in traditional wild yam chips processing to remove cyanogenic compounds. This review was based on the observations of traditional wild yam tuber chip processing and unstructured interview with the wild yam tuber chip maker at 6 locations in East Java, Indonesia. Relevant literature was used to explain the scientific basis of the detoxification methods based on the definite inclusion and exclusion criteria. Also, the variability of processing methods was compared among different locations. In general, the steps of traditional detoxification during wild yam tuber chips processing are slicing the peeled wild yam tubers, mixing with the rubbing ash, pressing, drying, soaking, boiling/steaming, and sun drying. Slicing, rubbing, and pressing in chips processing is aimed to convert cyanogenic glycoside into acetone cyanohydrin. The alkaline pH due to ash rubbing makes spontaneous decomposition of acetone cyanohydrin into HCN. HCN is easily removed by dissolution and heating (drying and steaming/boiling). Thermal treatment also spontaneously decomposes cyanohydrin into free HCN. All of the cyanogenic compounds are water-soluble which soaking and washing are aimed to remove all compounds. Consecutive, complicated, and time-consuming processing completely removes cyanogenic compounds and produces safe wild yam tuber chips. The key finding of this review is the purpose of every step in wild yam tuber detoxification has a scientific basis to reduce cyanogenic compounds gradually. This process produces a very low cyanide level in the final product. In conclusion, traditional detoxication reduces cyanogenic compounds to a safe level.

## Introduction

**Fig. 1:**
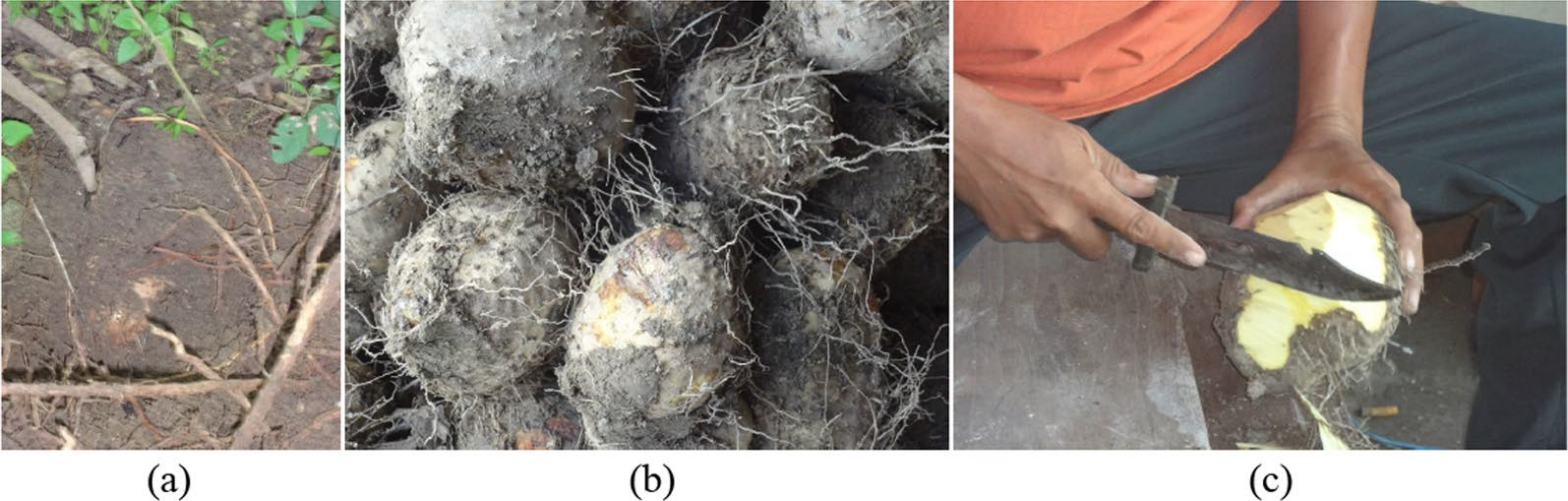
Wild yam (*Dioscorea hispida*) tuber in the soil under the stand of the forest (**a**), wild yam tubers with brown peel and covered by stiff roots (**b**), peeling the tuber before processing to remove the toxicant of cyanogenic compounds (**c**)

Indonesia has islands with tropical rainforests rich in biodiversity, including some tubers that grow wildly. The large forest in the past made forest is the source of foods, including tubers. The ancient people in Indonesia consumed cassava and consumed wild yam tuber as a staple food. This tuber grows in the soil under the stand of the forest and is used after peeling before processing to remove toxicant compounds. This tuber size is varied with the range of 10–25 cm in diameter. has brown peel, yellowish flesh, and covered by the stiff roots (Fig. 1).

During the time of Rumphius, a Dutch botanist in the era of Dutch colonialism, in 1830, several types of *Dioscoreaceae* were also eaten [1]. In the past, wild yam was widely consumed and almost all regions in Indonesia have local names for this tuber. Several tribes in Indonesia have the local name for this tuber such as Gadung (Sunda), Gadung (Jawa/Java), Gadung (Madura) at Java Island; Gadung (Bali) at Bali Island; Kapak (Sasak), Gadu (Bima), Iwi (Sumba) at Nusa Tenggara Province; Bitule (Gorontalo), Sikapa (Makasar), Salapa (Bugis), Ondo (Bitung) at Celebes Island; janèng (Aceh), janiang (Minangkabau) at Sumatera Island [2].

*Dioscoreaceae* family tubers are rich in bioactive compounds and also contain macro components such as starch, protein, fat, and dietary fiber [3]. The pronounced bioactive compounds of the yam family are diosgenin [4], dioscorin [5], and water-soluble polysaccharides [6]. Traditionally, diosgenin is a precursor in the pharmaceutical industry for hormonal drugs [4]. Diosgenin is a steroidal saponin that is effective as a therapeutic agent for several chronic diseases such as cancer, cardiovascular diseases, asthma, nervous system disorders, diabetes, arthritis, and others [7, 8]. Dioscorin (MW 31 kDa) is a yam tuber storage protein that accounts for 85% of total tuber protein [9]. This protein has biological activities as immunomodulatory [10], reducing oxidative stress and attenuating learning dysfunction [11], antihypertensive [12], and its derivative showed antioxidant and antiglycation activities [13]. Non-starch polysaccharide or mucilage of yams is one of the main bioactive compounds [14] that can reduce glucose blood level in hyperglycemia [6]. This mucilage also contains dioscorin as the bioactive protein [15]. Yam polysaccharide exhibits many important bioactivities such as immunomodulatory, hypoglycemic, antitumor, and antioxidant activities [14]. Due to the bioactive compounds, wild yam tuber is a candidate for functional food ingredients. Food ingredients and bioactive compounds are essential in supporting human health, including immune functions, specifically in preventing COVID-19 disease [16]. Undervalued bioresources [17], such as wild yam tuber, are considered sources of bioactive compounds and foods to support food security.

The disadvantage of wild yam tuber is the occurrence of toxic cyanogenic compounds. Bradbury et al. [18] revealed cyanogenic compounds composed of hydrogen cyanide (HCN), acetone cyanohydrin, and cyanogenic glycoside. The latest nitrile-containing compound is not toxic in intact form, but it produces toxic cyanide after enzymatic digestion. Consumption of this compound leads to cyanide poisoning [19]. Cyanogenic glycoside converts to acetone cyanohydrin with the assistance of the β-glycosidase enzyme, and this compound further degrades into free HCN [20]. Over-consuming of cyanogenic causes some symptoms of cyanide poisonings, such as dizziness, rapid respiration, vomiting, headache, mental confusion, and stupor [21].

In the past, wild yam was used mainly as the source of macro-components of food for energy supply. Although it contains cyanogenic compounds, the consumption of wild yam tuber has a long history. Ancient Indonesian people found ways to have safe and non-toxic tubers. Historically, in 1628, when Batavia (currently Jakarta) was under siege, people consumed cassava and wild yam as staple foods. This habit complemented the habit of consuming wild cassava from the forest, such as in Priangan (West Java) and parts of East Java. The consumption of rice as a staple food was not common at that time, and rice began to spread in 1800 AD. At that time, VOC soldiers who often kept to the villages often brought rice for their food. Rice was common until the first part of the nineteenth century, and tubers such as wild yam were common to consume during the Dutch colonial period [1].

In Nusa Tenggara and Maluku, wild yam tuber was commonly used as a staple food to substitute corn and sago, mainly in dry areas [14]. Traditional detoxification usually involves slicing and soaking. In Ambon, the wild yam tuber slices are manually kneaded in seawater and then soaked for 2–3 h until softened and then dried. In Aceh, wild yam tuber slices are put into the gunny sack and placed in the clean flowing water, such as a river, at least for 24 h, and then sundried. In Bali, after slicing, wild yam tuber is mixed with ash from the wood for traditional furnaces and then soaked in brine and washed by freshwater. The sun drying is conducted for 3 days. Another method in Kebumen, Central Java, after rubbing with the ash, the tuber was buried in the soil for 3–4 days and then washed with fresh water. The white milk color of the washing water indicates that the toxicant compounds are still present, which means the washing should be continued until the washing water is clear [1].

**Fig. 2:**
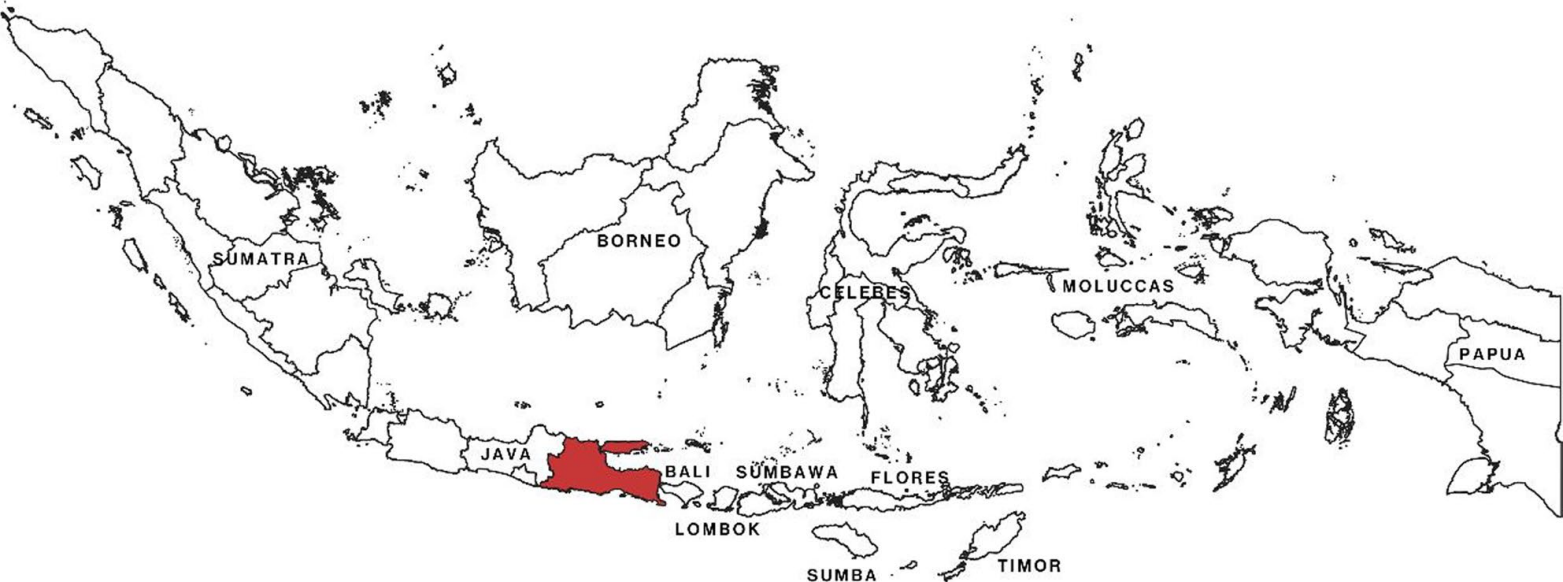
East Java province (red color) at Indonesian archipelago

In the 1980s, wild yam could be found in Indonesian markets, especially on Java Island, as wild yam tuber chips [1]. The chips are prepared traditionally to remove the toxic cyanogenic compounds by several steps, and there is a variability of preparation among places [22]. This paper aims to review the cyanogenic removal in every step of traditional wild yam tuber chips processing. The wild yam cultivation and the processing variability in East Java Province will also be discussed. East Java is one of the centers of wild yam tuber chips production (Fig. 2).

## Method of review

### Locations and observation

This review was based on the observation of the five locations of wild yam tuber chips makers at Kandangan Sub-district, Kediri Regency; Paiton Sub-district, Probolinggo Regency; Wates Sub-district, Kediri Regency; Ngaglik Sub-district, Blitar Regency; Ngluyu and Gamping Sub-districts, Nganjuk Regency; and Sawahan Sub-districts, Nganjuk Regency. These locations were selected randomly. The observation included the wild yam cultivation, cultivation locations, and chips tuber processing. The unstructured interview with the wild yam chips makers consisted of the wild yam chips processing, the purposes of processing steps, the equipment for chips production, and the marketing.

### Analysis of pH and cyanogenic compounds changes during processing

Four traditional detoxification methods from four locations (Kandangan Sub-district, Kediri Regency; Paiton Sub-district, Probolinggo Regency; Wates Sub-district, Kediri Regency; Ngaglik Sub-district, Blitar Regency) were conducted at the laboratory for analysis of the pH at every step in wild yam tuber chips processing. The cyanogenic changes during wild yam chips processing were from our previous study [22]. The discussion to elucidate the scientific evidence behind every step of wild yam tuber detoxification based on the relevant literature.

### Literature review

This literature review was conducted by selecting the relevant literature about the health benefits of the yam family, the bioactive compounds of yam, the history of wild yam consumption in Indonesia, the traditional wild yam detoxification in other regions in Indonesia, the cyanogenic-containing crops (such as cassava) detoxification methods, the characteristics of wild yam, the properties of cyanogenic compounds, detoxification methods applied for wild yam, and the cyanogenic compounds removal.

The literature was searched by using keywords of yam, wild yam, Dioscorea, dioscorin, diosgenin, water-soluble polysaccharides, cyanogenic compounds, cyanogenic glycosides, acetone cyanohydrin, HCN, ß glucosidase, hydroxyl nitrile liase (HNLase), tubers, chips, methods of cyanogenic compounds removal, from scholarly articles and scientific manuscripts.

The year of publication is from 1988 until 2022. All the studies of related scholarly manuscripts were a mixture of qualitative and quantitative data. The quantitative and qualitative data analysis followed the explanation in the cited articles and then discussed. Inclusion criteria were cyanogenic removal from food crops and their mechanism, and exclusion criteria were the removal of cyanogenic compounds from non-food materials.

## Wild yam cultivation

Wild yam is an inferior tuber that is consumed limitedly because this tuber consumption is associated with the poor [1]. However, the exploration of bioactive compounds and the scientific evidence of the safe level of cyanogenic compounds are expected to increase the consumer interest to consume these tuber-based products. Wild yam tuber chips are usually produced in the small household industries around the forest as the source of the main raw material of the chips. The wild yam tuber chips are sold in traditional markets around the location and nowadays are also sold limitedly in the modern markets.

**Fig. 3:**
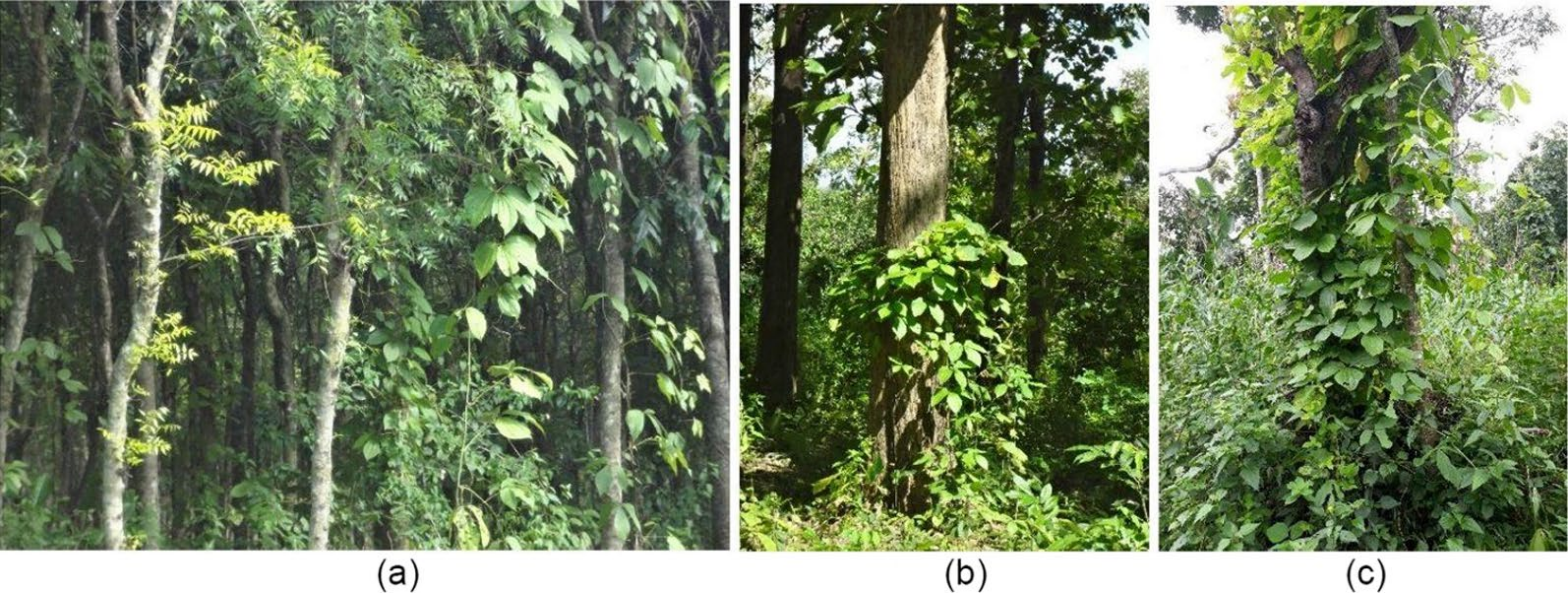
Wild yam plant under the stand of mahogany forest (**a**), teak forest (**b**), and community forest (**c**) at Nganjuk Regency, East Java, Indonesia

The tropical rainforest is the source of wild yam tuber. This tuber sometimes grows wildly and vines to the tree trunk (Fig. 3) and is rarely cultivated under the forest stand. In some places, the forest is overgrown with similar plants, such as mahogany (Fig. 3a) or teak (Fig. 3b), and the forest is managed under the national forest company (Perhutani). Some areas under Perhutani management in East Java are allowed to cultivate wild yam by the people around the forest at Nganjuk Regency (Fig. 3c). Wild yam cultivation is the livelihood of the community around the forest.

People around the forest process the wild yam tuber into chips. The tuber is not directly sold or used for foods due to a high cyanogenic compound content. Forest-based communities no longer consume wild yam tuber as a staple food, and the shifting staple food to rice makes the wild yam tuber is processed into chips. Local wisdom is used to produce wild yam tuber chips in removing cyanogenic compounds. The cyanogenic removal methods are preserved from generation to generation, and this community widely understands the knowledge about toxicant compounds from wild yam tuber.

## Traditional wild yam tuber chips processing

**Fig. 4:**
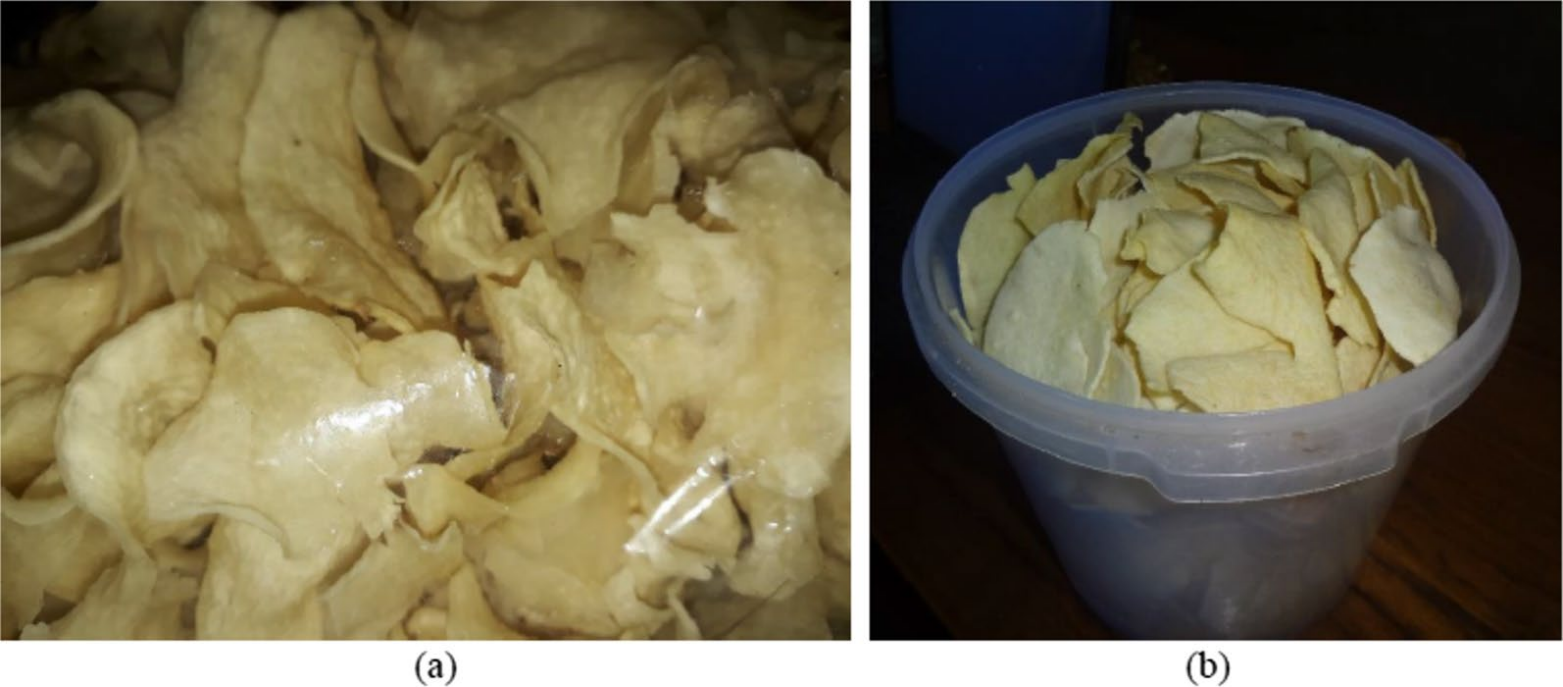
Processing of wild yam tuber into chips is complex and consisting of consecutive steps for detoxification to produce raw wild yam tuber chips (**a**) that are fried before consumption (**b**)

Processing in wild yam tuber chip-making is very complicated and time-consuming to remove cyanogenic compounds from wild yam tubers. However, this product price is quietly low due to the association of this inferior tuber for the poor people. The tuber chips processing survey at East Java revealed a slightly different process among chip makers from different locations. In general, the steps of cyanogenic compounds removal are slicing the peeled tubers, mixing with the rubbing ash, pressing, drying, soaking in the freshwater, steaming/boiling, and sun drying [19]. The raw wild yam tuber chips before frying are called “krecek” with a price of about US 2 per kg, and some are sold in the packaged fried chips (Fig. 4). The process of wild yam tuber chip processing is very complex compared to other tubers such as potatoes, which only involve peeling, washing, slicing, blanching, water draining, and frying [23].

**Fig. 5:**
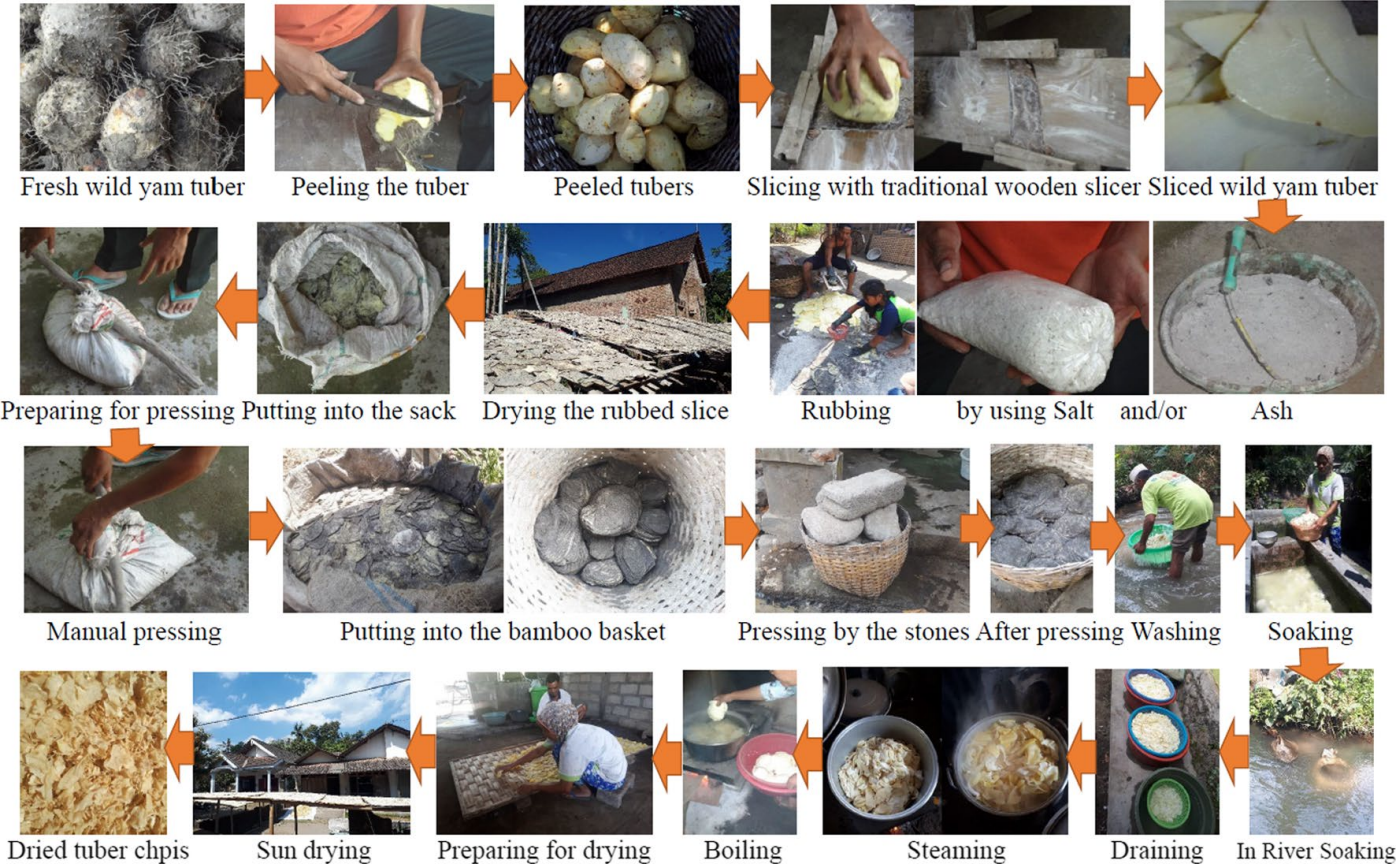
General steps for cyanogenic compounds removal in wild yam tuber chips preparation. The process consists of peeling the wild yam tuber; slicing by traditional wooden slicer; rubbing the sliced tuber by salt and/or ash; drying the rubbed sliced tuber; putting into sack and then manually pressing, or putting into bamboo basket and pressing by stones for a night; washing the pressed sliced tuber; soaking in the tub or soaking in the river; draining the soaked sliced tuber; steaming or boiling; and sun drying

The general steps of cyanogenic compounds removal are shown in Fig. 5. The tubers are manually peeled using a knife and then sliced with the traditional wooden slicer. The rubbing ash is prepared by mixing coarse salt with the wood ash from a traditional furnace. The sliced tubers are rubbed with a mixture of ash and/or salt until all the surface is covered. The type of rubbing agent is slightly different depending on the region. There is a mixture of ash and salt, salt only, or ash only. After rubbing, the sliced tubers are put into the sack for manually pressing. In some regions, the pressing is conducted by putting the big stones above the sack for a particular time, usually one night. Pressing aims to leach out the liquid from cell vacuoles. After pressing, the sliced tubers are soaked with fresh water, and frequently the water is replaced. The time for soaking and the frequency of water replacement are also varied among the regions. This step usually takes the longest time for about days. In the area that has a river with clear water, the sack containing sliced tubers is usually soaked in the flowing water of the river for days. After soaking, the sliced tubers are steamed or boiled for a particular time. The cooked sliced tubers are dried to remove water by sun drying. The dry chips are called “krecek” and are ready to fry before eating.

Observation of six locations of wild yam tuber chips makers at East Java (2 locations at Kediri Regency but different sub-district, Blitar, and Probolinggo Regency. Two locations at Nganjuk Regency with different Sub-district) showed that the general steps of cyanogenic compounds removal are similar. However, there are some differences mainly in the rubbing materials, duration of soaking and pressing, and the methods of gelatinization by steaming or boiling. The pressing methods are by putting the big stone above the woven bamboo basket or by putting the rubbed tuber slices into the sack and then manually pressing with an assistance of a wood stick. The latter method is found at Ngluyu and Gamping Sub-districts, Nganjuk Regency, around the teak and mahogany forest. However, in the same regency but different sub-district at Sawahan, Margopatut Village, the removal of cyanogenic compounds does not involve pressing. After slicing, the sliced tubers are rubbed with salt to leach out the mucilage and then soaked in the brine for 3 days. The local wisdom of cyanogenic removal is very region-specific and hereditary. The differences in wild yam tuber chips processing at East Java are shown in Table 1.

**Table 1:** Variability of wild yam tuber chips processing at East Java, Indonesia

Step	Kandangan Sub-district, Kediri Regency [19]	Paiton Sub-district, Probolinggo Regency [19]	Wates Sub-district, Kediri Regency [19]	Ngaglik Sub-district, Blitar Regency [19]	Ngluyu and Gamping Sub-districts, Nganjuk Regency	Sawahan Sub-districts, Nganjuk Regency
Peeling	Manual	Manual	Manual	Manual	Manual	Manual
Slicing	Manual	Manual	Manual	Manual	Manual and Mechanical	Manual
Rubbing	Wood ash only	Wood ash: salt 25:1 (w/w), Stand for 1 h	Wood ash: salt 50:1 (w/w)	Wood ash: rice straw ash 2:1 (w/w) ash: salt 5:1 (w/w)	Wood ash and salt (the ratio is not observed)	Salt
Pressing	By stone for 12 h	By stone for 12 h	By stone for 12 h	By stone for 12 h	Manual by using gunny sack and wooden stick	Not applicable
Drying	Sun drying 1–3 h	Sun drying 1–3 h	Sun drying 1–3 h	Sun drying 1–3 h	Not applicable	Not applicable
Washing	By flowing water at the river	By freshwater	By freshwater	By freshwater	By freshwater	Not applicable
Soaking	36 h	12 h	48 h	36 h	3 days	3 days
Pregelatinization	Boiling 30 min	Boiling 15 min	Boiling 15 min	Steaming 30 min	Steaming until cooked	Steaming until cooked
Drying	Until dry	Until dry	Until dry	Until dry	Until dry	Until dry

People in the area surrounding the forest are doing the toxicant compounds removal from wild yam tubers based on the experience. The proper removal indicator is traditionally checked by observing the leached liquid from the sliced tuber. The white milk or yellowish color means the cyanogenic compounds are still present, and the process is improper. Another method is by checking the bitterness of the sliced tubers. Cyanogenic compounds are associated with a bitter taste, and this process is not always correct. Thus, wild yam tuber chips poisoning sometimes occurs. It is a challenging to apply a simple method of cyanide level check, such as using a test kit paper [24], to check the complete removal of toxicant compounds.

## Cyanogenic compounds removal in the steps of wild yam tuber chips processing

The main problem of cyanogenic compounds removal of wild yam tubers is the existence of these toxicant compounds in the cell vacuoles [25]. The cyanogenic glycoside is not toxic on its own. Disruption of the cells structures of tubers leads to contact of the β glucosidase enzyme with its substrate, cyanogenic glycoside. Hydrolysis of cyanogenic glycoside by this enzyme produces sugar and cyanohydrin [26]. Subsequently, cyanohydrin is catalyzed by the hydroxy nitrile lyase (HNL) enzyme, which is also found in cyanogenic plants [27], that rapidly degrade into HCN and ketones or aldehydes [26], or spontaneously HCN is released at pH > 5 [28]. The formation of HCN is the main cause of the acute toxicity of cyanides. The boiling point of HCN is 25.6 °C which makes HCN quickly evaporate in the drying [29] or heating process. Cyanogenic glycoside is a water-soluble compound [30], and the main functions of cyanogenic glycoside removal are by water dissolution and conversion of this compound into HCN that is easily removed by heating or drying.

Actually, the traditional wild yam tuber detoxification in chips processing is aimed to remove cyanogenic glycoside by water solubilization, converting this compound into free HCN and HCN removal by drying and heating. Consecutive and long-time preparation is required because of the high cyanogenic compounds level in wild yam tuber. HCN content of wild yam tuber was 84.26 ppm [31], and total cyanide content of 379–739 ppm [32]. The previous study [22] showed that wild yam tuber contained HCN of 54.74 ± 0.17 ppm, acetone cyanohydrin of 3.91 ± 0.28 ppm, a cyanogenic glycoside of 24.55 ± 0.21 ppm, and total cyanides of 83.20 ± 0.11 ppm. FAO/WHO determined that the safe limit of cyanide ingestion is 10 ppm [33]. A simple and rapid method to check the cyanide level of wild yam tuber chips is an important way to assure the safety of the final product. Many rapid methods to control toxicants or contaminants have been designed and tested [34]. The promising rapid method for cyanide level detection in wild yam tuber chips is a paper-based kit developed by Malahom et al. [24].

The followings are the review of each step in cyanogenic compounds removal in wild yam tuber chips processing.

### Slicing

The first step of cyanogenic compounds removal after peeling is slicing. Some of the tuber cells are disrupted during slicing, allowing the *β* glucosidase enzyme from cell walls to contact the cyanogenic glycoside from the ruptured vacuoles. According to Siritunga and Sayre [35], linamarase (*β* glucosidase enzyme in cassava) is found in the cell walls. The hydrolysis of a cyanogenic glycoside is started by disrupting the tuber tissue to release endogenous enzymes [36].

During slicing, the ruptured cells are restricted, and the disrupted cells will be more pronounced if the tuber tissue is grated or ground. Grating and crushing are tissue disintegration which usually removes cyanides very efficiently because the plant cells are completely broken down [37]. Ruptured cells allow contact between the β glucosidase enzyme and its substrate directly, resulting in the liberation of acetone cyanohydrin. HCN and acetone are easily liberated spontaneously from acetone cyanohydrin in alkaline pH, and then HCN is discarded by evaporation to air or dissolving into water. Flowing water is more efficient to wash out the HCN, besides the intact cyanogenic glycoside and acetone cyanohydrin also dissolve well in the water. Washing of the sliced tubers in the flowing water is found at Kandangan Sub-district, Kediri Regency (Table 1).

Our previous study [38] showed that removal of cyanogenic compounds from bitter cassava tuber was conducted by grating the tuber flesh before spontaneously fermenting. Combination with fermentation decreases the cyanides level to 90–93%. However, cell disruption by grating or grinding is not applicable for wild yam tuber chips processing. During slicing, the conversion of cyanogenic glycoside into cyanohydrin might take place to some extent. The degree of cyanogenic glycoside conversion might be affected by several factors such as the degree of cell disruption, the thickness of the slice, and the duration of delay time to the next steps.

### Ash/salt rubbing

After slicing, the next step is ash/salt rubbing into the surface of tuber slices. Traditionally, it aims to leach out more of the liquid from the tuber tissue, and ash/salt increases solid concentration around the tissue and causes more liquid to leach out from the cells and vacuoles. The type of ash also varies between chip makers, most of them use wooden ash, but there is also the use of the mixture of wood ash and rice straw (Table 1). CaO dominates the mineral composition of the rubbing agent of ash and salt, and rice straw contributes to the concentration of SiO_2_ [22]. The minerals from the ash increase the pH of the sliced tubers (Table 2).

**Table 2:** Changes of the pH during traditional wild yam tuber chips processing

Processing Step	Kandangan Sub-district, Kediri Regency	Paiton Sub-district, Probolinggo Regency	Wates Sub-district, Kediri Regency	Ngaglik Sub-district, Blitar Regency
Fresh tuber	6.63 ± 0.15	7.23 ± 0.60	6.63 ± 0.42	6.53 ± 0.49
Rubbing (after 1 h)	9.23 ± 0.25	8.80 ± 0.46	9.53 ± 0.25	9.50 ± 0.20
Pressing	7.77 ± 0.32	8.10 ± 0.72	8.57 ± 0.47	8.87 ± 0.31
Drying	7.97 ± 0.21	8.97 ± 0.21	8.97 ± 0.42	7.63 ± 0.42
Washing	8.63 ± 0.25	8.17 ± 0.32	8.57 ± 0.47	7.77 ± 0.51
Soaking	6.53 ± 0.42	6.97 ± 0.15	7.57 ± 0.35	7.23 ± 0.51
Soaking water	9.00 ± 0.46	7.27 ± 0.15	8.97 ± 0.31	8.00 ± 0.30
Boiling/steaming	7.77 ± 0.42	7.37 ± 0.51	7.53 ± 0.45	7.87 ± 0.38
Drying	8.23 ± 0.90	7.60 ± 0.36	7.27 ± 0.31	8.10 ± 0.56

Primary data from three replications

The pH changes of during wild yam chips processing at Nganjuk Regency were not measured

The pH changes of during wild yam chips processing at Nganjuk Regency were not measured.

Alkaline pH triggers the decomposition of acetone cyanohydrin into free HCN and acetone. Decomposition is optimum at pH > 5 and temperature above 35 °C [39]. The next step that involves heating or water dissolution is required to reduce HCN from the sliced tubers. The presence of HNLase in the wild yam tubers is still unknown. In the cassava, the accumulation of acetone cyanohydrin is found after cyanogenic glycoside hydrolysis by *β* glucosidase enzyme. Cassava root lacks HNLase enzyme because this enzyme is only found in the leaves, thus making the main obstacle of cyanide removal of cassava [39].

The soaking time of rubbed slices affected the degree of cyanide reduction. The highest reduction was found in the longest time of 36 h which the cyanide acid was reduced to 51.42% [40]. The time for rubbing ash to interact with the surface of sliced tuber in the traditional methods is only 1 h. Delay time for 1 h (Table 1) lets the vacuole fluid leach out. That time is not sufficient to reduce cyanides level significantly from the tubers. The tuber chips maker is aware of this problem, and the next long steps are required.

Rubbing with only salt in the method of the wild yam tuber chip maker at Sawahan Sub-district, Nganjuk Regency, does not increase the pH because only using salt. The acetone cyanohydrin might accumulate in the sliced tubers. After rubbing, the sliced tubers are soaked in the brine for 3 days to remove this compound and the remaining cyanogenic glycoside. The soaking water is repeatedly replaced to avoid the saturation of cyanogenic compounds. The effectiveness of this method compared to others is still unknown, but this method has not been reported to cause cyanide poisoning.

### Pressing and drying

After rubbing sliced tubers standing for 1 h, the next step to remove cyanides is pressing. There are two pressing methods (Table 2): putting the big stone above the basket containing rubbed sliced tubers or pressing manually (Fig. 5). Pressing by using stones requires a long time, usually one night. The basket is let with the pressure from the stones. Manual pressing uses a wooden stick rotated manually, which raises a compressive force. The disadvantage of the latter method is the inconsistent pressure force because of using human power.

The pressing is aimed to leach out the cell fluid more, including the alkaloid containing cyanogenic glycoside from vacuoles. The high concentration of the ash/salt in the surface of sliced tubers makes the concentration difference between outside and inside tuber cells. The fluid from the cells will leach out to balance the concentration outside. The ruptured cell walls will release *β* glucoside enzyme, and the contact of this enzyme with cyanogenic compounds in the leached liquid leads to the hydrolysis of this compound. Acetone cyanohydrin and glucose are liberated in this process. The alkaline pH caused by the presence of the ash (except if the rubbing only uses salt) will spontaneously decompose cyanohydrin into HCN. HCN, acetone cyanohydrin, and cyanogenic glycosides will dissolve in the soaking water in the next step. High residual acetone cyanohydrin in the cassava was attributed to the low-pH during soaking (fermentation) [39]. The experience guides the wild yam tuber chips makers to use ash to reduce cyanogenic compounds. This traditional method is passed down from generation to generation and is still maintained.

The pH values decrease in four methods during pressing (Table 2). The fresh tuber has a slightly acidic pH that might be related to the organic acids in the cell vacuoles. The ruptured cells secrete acid-containing fluid, and the neutralization reaction might take place during this step. The possibility of microbial growth during this process is relatively low, because the time is short, the concentration of ash/salt is high, and the pH is alkaline, which does not support the microbial growth. After pressing, the sliced tubers are watery because of the leached liquid from the tuber cells.

During subsequent sun drying for 1–3 h, the acetone cyanohydrin decomposition into HCN and acetone is possible. HCN is easily removed by heating or washing. Acetone cyanohydrin decomposes at a temperature above 35 °C or pH above 5 [39]. In tropical regions, the air temperature during sun drying is about 40 °C. In this way, some HCN evaporates, but because of short duration, presumably, not all of the cyanogenic glycoside has been converted into cyanohydrin, which makes additional long steps still required.

### Washing and soaking

Washing is aimed to discard the remaining ash and the leached fluid from the cells. Washing is using freshwater, and the process is stopped when the viscous liquid is no longer present. Another method uses flowing water at the river (Table 1). The flowing river is more effective in washing the viscous liquid from the surface of sliced tubers, and it is also effective in reducing cyanogenic compounds. In the past, one of the ways to detoxify wild yam tubers was soaking at the river for days. The pH after washing generally increases compared to the pressing step (Table 2). Some minerals might be absorbed during pressing, resulting in a pH increase. This condition is beneficial to decompose cyanohydrin into free HCN due to the high pH.

Soaking is carried out by putting the sliced tubers in the tubs and the freshwater is added until all the surface of the slices is covered. Another method at Sawahan Sub-district, Nganjuk Regency, the soaking method is carried out in the brine. Soaking with seawater for 12–36 h reduced the cyanide level by 48–71% [40]. In all methods, the soaking water is frequently replaced by new freshwater. This process is aimed to remove the leached cyanogenic compounds effectively. Soaking water replacement by freshwater increases the solubility of all cyanogenic compounds by preventing the saturated solution. During soaking, cyanogenic compounds are dissolved and also other soluble components such as protein, soluble fibers, and starch.

The possibility of spontaneous fermentation by microbial growth during soaking is low because the frequent replacement by freshwater prevents the microbes from growing. Our previous study [41] showed that submerged fermentation of high cyanide cassava induced microbial growth after 3 days of soaking. Some studies reported that soaking wild yam tubers also reduced the cyanogenic compounds [31, 40]. Leaching with different flowing water rates reduced cyanide levels of wild yam in the range of 29–51%. Meanwhile, subsequent steaming after leaching increased the cyanide reduction by 35% [31]. Soaking in seawater for 12, 24, and 36 h reduced the cyanide level of sliced wild yam tuber 48.30%, 64.44%, and 70.88% [40]. During soaking, the water was replaced every 3 h. Meanwhile, soaking of sliced wild yam tuber with rubbing ash for 12, 24, and 36 h decreased cyanide levels by 24.09%, 38.69%, and 51.42%. Based on that study [40], soaking with rubbing ash was less effective than seawater soaking, presumably related to the high concentration of rubbing ash using 75% of sliced tuber weight. In traditional wild yam chips processing, the rubbing ash is washed prior to soaking, which might avoid a high concentration of soaking water that will inhibit the diffusivity of cyanogenic compounds from the cell vacuoles.

### Steaming or boiling

After soaking, the sliced tubers are drained and boiled or steamed. In this step, the HCN released from the previous steps is eliminated by heating. Residual acetone cyanohydrin also spontaneously decomposes due to the temperature above 35 °C, and HCN has a boiling point of 25.6 °C and easily evaporates during steaming and boiling. During this heating, starch is gelatinized, and protein is denatured. This step makes a uniqueness of the chips due to starch pregelatinization that results in chips expanding easily during frying.

### Drying

Drying is the final step in the consecutive and long cyanide removal in wild yam tuber chips processing. The main goal of drying is to have dry raw chips or “krecek” that have a long shelf life. Some of the “krecek” is sold in bulk in traditional markets, and some are fried to sell in packaged fried chips. Drying needs time for days depending on the weather. Usually, the wild yam tuber chips makers are located around the forest in the highland area of mountains or volcanoes where the sunlight temperature is not too high. Another problem during sun drying is the mold attack if the weather is not sunny for days.

The residual HCN and acetone cyanohydrin are removed during drying, but cyanogenic glycoside cannot be evaporated. Therefore, the previous steps should properly convert the cyanogenic glycoside into acetone cyanohydrin and then free HCN. Cyanogenic glycoside could not be decomposed by heat treatment. The cyanogenic glycoside toxicity depends on the spontaneous HCN release or enzymatic hydrolysis. The toxicity of cyanogenic glycoside is estimated based on the quantity of generated free cyanide from hydrolysis [42]. The main problem of wild yam tuber is that cyanogenic glycoside is the predominant cyanogenic compound [22].

## Degree of cyanogenic compounds removal

The main problem in cyanide detoxification is the presence of cyanogenic glycoside that is not easy to remove by heating. Therefore, in wild yam tuber chips processing, many steps are required, such as combining cell fluid leaching, adjusting alkaline pH, pressing, washing, soaking, boiling/steaming, and drying. The single step to reduce cyanide from wild yam tuber is not applicable, and several consecutive steps are required to remove cyanides in the tuber chips altogether. Table 3 shows the reduction in cyanogenic compounds after processing wild yam chips.

**Table 3:** Cyanogenic compounds removal during wild yam tuber chips processing

Location	Cyanogenic glycosides		Acetone cyanohydrin		Free HCN		Cyanogenic compounds	
	ppm	Reduction (%)	ppm	Reduction (%)	ppm	Reduction (%)	ppm	Reduction (%)
Fresh yam tuber	54.74 ± 0.17		3.91 ± 0.28		24.55 ± 0.21		83.20 ± 0.11	
Kandangan Sub-district, Kediri Regency	0.34 ± 0.05	99.38	0.10 ± 0.05	97.44	1.32 ± 0.04	94.62	1.76 ± 0.28	97.88
Paiton Sub-district, Probolinggo Regency	0.45 ± 0.02	99.18	0.13 ± 0.04	96.68	1.33 ± 0.04	94.58	1.95 ± 0.16	97.66
Wates Sub-district, Kediri Regency	0.49 ± 0.03	99.10	0.13 ± 0.03	96.68	1.35 ± 0.02	94.50	1.97 ± 0.09	97.63
Ngaglik Sub-district, Blitar Regency	0.53 ± 0.09	99.03	0.36 ± 0.06	90.79	1.36 ± 0.06	94.46	2.25 ± 0.42	97.30

Reference [22]

Table 3 shows that the reduction in the most difficult compound to remove, cyanogenic glycoside, by traditional detoxification in wild yam tuber chips processing achieved 99%, which mean this compound is almost completely removed. The presence of cyanogenic glycoside is the main obstacle of wild yam tuber utilization. The removal of HCN and cyanohydrin is lower than cyanogenic glycoside, probably due to the remaining HCN and cyanohydrin that do not evaporate during drying. The thickness of the slices might affect the degree of cyanogenic compound removal. The thin tuber slices have more ruptured than the thick slices cells, and the cell liquid is easier to leach out. Overall, the traditional detoxification could reduce about 97% of cyanogenic compounds, and the level of remaining cyanides is safe to consume.

The detoxification process during wild yam tuber chips processing is very complicated and time-consuming. The combination of several methods is required to have almost proper cyanide removal. The main obstacle of cyanide removal, the presence of cyanogenic glycoside, by simultaneous steps is converted into acetone cyanohydrin and HCN, which are easier to remove. The cyanogenic compounds could not be successfully removed if the process is only one step or a combination of two methods. Many studies showed that a simple and single method to eliminate cyanogenic compounds did not produce satisfactory results. Heap fermentation (drying followed by fermentation) reduces total cyanides one eighth because some cells remain intact [43]. During heap fermentation, the cyanogenic glycoside is decomposed by microbes [44, 45]. However, boiling treatment is inefficient due to the inactivation of *β* glucosidase, but HCN is more effectively reduced by boiling than steaming, roasting, or frying. Pressing after grating or fermentation could decrease 70–95% free HCN [46].

## Conclusion

Traditional detoxification during wild yam tuber chips processing aims to convert cyanogenic glycosides into acetone cyanohydrin and then decompose to free HCN. Cyanogenic glycoside, acetone cyanohydrin, and HCN are water-soluble. Therefore, traditional detoxification involves soaking and washing. The first step in traditional detoxification is slicing, which might rupture the tubers' cell tissue. Disrupted cells and liquid leaching from vacuoles lead to the contact between *β* glucosidase enzyme with cyanogenic glycoside to release sugar and cyanohydrin. This process is exaggerated by ash rubbing and pressing using big stone or manually. Pressing is also aimed to leach out liquid from cell vacuoles. Drying after pressing make the *β* glucosidase have sufficient time to hydrolyze cyanogenic glycoside, and the resulting acetone cyanohydrin spontaneously releases HCN due to the alkaline pH from ash rubbing. Washing step in the flowing water such as the river or repeatedly washing removes the cyanogenic compounds in the surface of the sliced tubers. Soaking is usually conducted in freshwater, and some use brine. Frequent replacement by freshwater is to make sure that the solubility of cyanogenic compounds is maintained because the saturation of the soaking water is prevented. Heating above 35 °C spontaneously decomposes cyanohydrin into HCN, and HCN is easily removed by high temperature because it has a low boiling point. Drying after steaming/boiling, besides evaporating water to have dry chips, also aims to evaporate residual HCN and cyanohydrin. Consecutive, complicated, and time-consuming detoxification in tuber chip processing results in almost complete cyanogenic removal, and the chips are safe to consume.

The limitation of this review is the discussion focused on the detoxification methods at six locations in East Java. The selected locations were not representing all regencies in this province. Generally, the wild yam tuber chip makers are found in almost every area around the forest. The standardized process is required to assure that the finished product of wild yam tuber chips is safe to consume and has cyanogenic compounds below 10 ppm. The wild yam chip makers should be equipped with the knowledge to check cyanide levels simply using a practical kit. Simply practical kits with an affordable price and easy to obtain should be developed. Local wisdom in detoxification principles of wild yam tuber should be maintained but made more efficient and modern through process mechanization and control. The role of government is very important to empower the wild yam tuber chip makers. The scholars should have a role to prove all the traditional wild yam tuber detoxifications is safe and elucidating the mechanism of cyanides reduction scientifically.

## Data Availability

Not applicable.
